# Can Novel Management Practice Improve Soil and Environmental Quality and Sustain Crop Yield Simultaneously?

**DOI:** 10.1371/journal.pone.0149005

**Published:** 2016-02-17

**Authors:** Upendra M. Sainju

**Affiliations:** USDA-ARS, Northern Plains Agricultural Research Laboratory, Sidney, MT 59270, United States of America; Chinese Academy of Sciences, CHINA

## Abstract

Little is known about management practices that can simultaneously improve soil and environmental quality and sustain crop yields. The effects of novel and traditional management practices that included a combination of tillage, crop rotation, and N fertilization on soil C and N, global warming potential (GWP), greenhouse gas intensity (GHGI), and malt barley (*Hordeum vulgarie* L.) yield and quality were examined under non-irrigated and irrigated cropping systems from 2008 to 2011 in eastern Montana and western North Dakota, USA. In loamy soil under non-irrigated condition in eastern Montana, novel and traditional management practices were no-till malt barley-pea *(Pisum sativum* L.) with 80 kg N ha^-1^ and conventional till malt barley-fallow with 80 kg N ha^-1^, respectively. In sandy loam soil under irrigated and non-irrigated conditions in western North Dakota, novel and traditional management practices included no-till malt barley-pea with 67 (non-irrigated) to 134 kg N ha^-1^ (irrigated) and conventional till malt barley with 67 (non-irrigated) to 134 kg N ha^-1^ (irrigated), respectively. Compared with the traditional management practice, soil organic C (SOC) and total N (STN) at 0–120 cm were 5% greater with the novel management practice under non-irrigated condition in eastern Montana and under irrigated condition in western North Dakota, but were not different under non-irrigated condition in western North Dakota. In both places under irrigated and non-irrigated conditions, total applied N rate, residual soil NO_3_-N content at 0–120 cm, global warming potential (GWP), and greenhouse gas intensity (GHGI) were 15 to 70% lower with the novel than the traditional management practice. Malt barley yield and quality were not different between the two practices in both places. Novel management practices, such as no-till malt barley-pea with reduced N rate_,_ can simultaneously enhance soil and environmental quality, reduce N input, and sustain crop yield compared with traditional practices in the northern Great Plains, USA.

## Introduction

Traditional practices, such as conventional till with crop-fallow or continuous monocropping with N fertilization, have reduced soil C and N storage and crop yields due to residue incorporation into the soil, aggregate disruption, and absence of crops during fallow in the northern Great Plains [1, 2]. Residue incorporation into the soil increases its contact with microorganisms, thereby increasing its mineralization and reducing soil C and N storage. Both tillage and fallow can increase soil organic matter mineralization due to increased microbial activity, thereby increasing residual soil NO_3_-N content and the potential for N leaching [2, 3]. Continuous monocropping can reduce crop yields compared with crop rotation due to increased infestation of weeds, diseases, and pests [4]. Traditional practices can also increase net greenhouse gas (GHG) emissions compared with improved practices, such as no-till with crop rotation and reduced N rate [5, 6].

Improved management practices, such as no-till, diversified crop rotation, increased cropping intensity, cover cropping, and reduced rate of N fertilization, can increase soil C and N storage and reduce GHG emissions compared with traditional practices [1, 7, 8, 9]. No-till can increase soil organic C (SOC) and soil total N (STN) and reduce GHG emissions compared with conventional till by reducing soil disturbance, residue incorporation, and microbial activity, which in turn, lower CO_2_ emissions [6, 10, 11]. Increased quality and quantity of crop residue returned to the soil from increased cropping intensity, diversified crop rotation, cover cropping, and N fertilization can increase SOC and STN compared with fallow, monocropping, and no cover crop or N fertilization [6, 12, 13]. Nitrogen fertilization stimulates N_2_O emissions [6, 14, 15], but has a variable effect on CO_2_ and CH_4_ emissions [13, 16, 17, 18]. Therefore, practices that can reduce N fertilization rates without influencing crop yields can substantially lower net global warming potential (GWP) and greenhouse gas intensity (GHGI) [6, 19].

No-till can reduce crop residue and soil organic N mineralization and residual soil NO_3_-N content which is subjected to leaching in the groundwater compared with conventional till [3]. Most crops are unable to uptake NO_3_-N from subsurface soils unless they have deep root systems [20]. Soil profile NO_3_-N content can be reduced with increased cropping intensity due to greater N immobilization, less summer fallow, and greater amount of N removed by crops [20]. Nitrogen loss below the root zone can be enhanced due to increased fallow period as a result of leaching and absence of crops to utilize N [3].

Soil water conservation is also greater in no-till than conventional till [21]. As a result, crop yields can be sustained by utilizing soil water more efficiently in no-till systems [2, 21, 22, 23]. Inclusion of pea in rotation with spring wheat (*Triticum aestivum* L.) and barley can sustain their yields by efficiently utilizing soil water and reduce N fertilization rate due to N supplied from pea residue which is rich in N as a result of biological N fixation from the atmosphere [4, 24, 25]. In addition, pea uses less soil water than spring wheat and barley, thereby leaving more water available for succeeding crops and increasing their yields [4, 26]. Other benefits of crop rotation compared with monocropping include decreased pressure from weeds, diseases, and pests [4, 27], reduced chemical inputs, and improved economic and environmental sustainability [28].

Although information on the effects of tillage, cropping systems, and N rates on soil C and N and crop yields in the northern Great Plains is available [13, 23], little is known about novel management practices that can simultaneously enhance soil organic matter, reduce N rate, potential for N leaching, and GHG emissions, and sustain crop yields and quality in the northern Great Plains, USA. This study compared novel and traditional management practices that included combinations of tillage, cropping systems, and N fertilization rates on soil C and N, GWP, GHGI, and malt barley yield and quality under non-irrigated and irrigated conditions from 2008 to 2011 in eastern Montana and western North Dakota, USA. It was hypothesized that the novel management practice (no-till barley-pea with reduced N rate) can increase soil C and N storage, reduce residual soil NO_3_-N content, GWP, and GHGI, and sustain malt barley yield and quality compared with the traditional management practice (conventional till malt barley-fallow or continuous malt barley with recommended N rate).

## Materials and Methods

The details of the experiment were described by Sainju et al. [29]. The studies were conducted on government farms owned by USDA-ARS, Sidney, MT and no specific permission was needed to conduct the study and collect data. Also, the field study did not involve protected or endangered species. Briefly, the experiments were conducted under non-irrigated condition from 2006 to 2011 in Sidney, eastern Montana and under irrigated and non-irrigated conditions from 2005 to 2011 in Nesson Valley, western North Dakota, both were located in the northern Great Plains, USA. In Sidney, mean monthly air temperature ranges from -8°C in January to 33°C in August and mean (68-yr average) annual precipitation is 340 mm. The soil was Williams loam (fine-loamy, mixed, frigid, Typic Argiborolls) with 350 g kg^-1^ sand, 325 g kg^-1^ silt, 325 g kg^-1^ clay, 11.5 g kg^-1^ SOC, and 7.2 pH at the 0–15 cm depth. In Nesson Valley, mean monthly air temperature ranges from -5°C in January to 32°C in August and mean (56-yr average) annual precipitation is 405 mm. The soil was Lihen sandy loam (sandy, mixed, frigid, Entic Haplustoll) with 720 g kg^-1^ sand, 120 g kg^-1^ silt, 160 g kg^-1^ clay, 11.2 g kg^-1^ SOC, and 7.7 pH at the 0–15 cm depth.

In Sidney, treatments included a combination of four tillage and cropping sequences (no-till continuous malt barley, no-till malt barley-pea, no-till malt barley-fallow, and conventional till malt barley-fallow), each with a split-plot treatment of four N rates (0, 40, 80, and 120 kg N ha^-1^). In Nesson Valley, treatments included two irrigation systems (irrigated vs. non-irrigated), each with a split-plot treatment of five management practices (conventional till malt barley with 67 or 134 kg N ha^-1^, conventional till malt barley with 0 kg N ha^-1^, no-till malt barley-pea with 67 or 134 kg N ha^-1^, no-till malt barley with 67 or 134 kg N ha^-1^, and no-till malt barley with 0 kg N ha^-1^). The recommended N rates for irrigated and non-irrigated malt barley in Nesson Valley were 134 and 67 kg N ha^-1^, respectively. Actual amount of N fertilization rates applied to malt barley, however, varied from year to year (Tables 1–3), because N rates were adjusted to soil NO_3_-N content to a depth of 60 cm that was measured after crop harvest in the fall of the previous year. No N fertilizer was applied to pea. Both experiments were arranged in randomized complete block design with three replications. For this study, only no-till malt barley-pea with 80 kg N ha^-1^ as the novel management practice and conventional-till malt barley-fallow with 80 kg N ha^-1^ as the traditional management practice were selected in Sidney. Under non-irrigated condition in Nesson Valley, only no-till malt barley with 67 N ha^-1^ as the novel management practice and conventional till malt barley with 67 kg N ha^-1^ as the traditional practice were selected. Similarly, under irrigated condition in Nesson Valley, only no-till malt barley with 134 N ha^-1^ as the novel management practice and conventional till malt barley with 134 kg N ha^-1^ as the traditional practice were selected.

**Table 1:** Comparison of novel (no-till malt barley-pea with 80 kg N ha^-1^) and traditional (conventional till malt barley-fallow with 80 kg N ha^-1^) management practices on ecosystem services under non-irrigated condition in Sidney, eastern Montana.

Parameter	Novel management practice					Traditional management practice					Difference†
	2008	2009	2010	2011	Mean	2008	2009	2010	2011	Mean	
Soil organic C (Mg C ha^-1^)‡	180	182	185	183	183	178	176	180	166	175	8 (5)*
Soil total N (Mg N ha^-1^)‡	16.0	16.8	17.3	17.1	16.8	15.8	16.6	17.0	16.6	16.5	0.3 (2)
Residual soil NO_3_-N (kg N ha^-1^)‡	75	90	95	60	80	82	95	95	132	101	-21 (-21)**
Total N rate (kg N ha^-1^)§	60	58	49	43	210	57	49	53	23	182	28 (15)
GWP (kg CO_2_ eq. ha^-1^ yr^-1^)§	188	-124	-125	541	120	565	350	124	573	403	-283 (-70)***
GHGI (kg CO_2_ eq. kg^-1^ grain yield)¶	0.08	-0.05	-0.05	0.22	0.05	0.23	0.14	0.05	0.18	0.15	-0.10 (-67)***
Barley grain yield (Mg ha^-1^)	2.40	2.55	2.75	2.50	2.55	2.45	2.50	2.72	2.85	2.63	-0.08 (-3)
Barley grain concentration (g kg^-1^)	127	145	140	128	135	129	140	135	132	134	1 (1)
Barley grain plumpness (g kg^-1^)	768	770	750	800	772	710	758	687	793	737	35 (5)*

*Significant at *P* = 0.05

**Significant at *P* = 0.01

***Significant at *P* = 0.001

† Number in parenthesis denotes value in percentage.

‡ Measured at the 0- to 120-cm soil depth.

§ Global warming potential

¶ Greenhouse gas intensity

**Table 2:** Comparison of novel (no-till malt barley-pea with 67 kg N ha^-1^) and traditional (conventional till malt barley with 67 kg N ha^-1^) practices on ecosystem services under non-irrigated condition in Nesson Valley, western North Dakota.

Parameter	Novel management practice					Traditional management practice					Difference†
	2008	2009	2010	2011	Mean	2008	2009	2010	2011	Mean	
Soil organic C (Mg C ha^-1^)‡	53	59	65	67	61	54	61	67	70	63	-2 (-3)
Soil total N (Mg N ha^-1^)‡	9.0	9.7	10.5	10.0	9.8	9.3	9.9	10.8	10.4	10.1	-0.3 (-3)
Residual soil NO_3_-N (kg N ha^-1^)‡	25	40	53	22	35	51	60	75	78	66	-31 (-47)**
Total N rate (kg N ha^-1^)§	30	25	20	30	105	50	40	35	35	160	-55 (-34)
GWP (kg CO_2_ eq. ha^-1^ yr^-1^)§	503	795	324	362	496	1501	2005	1121	1145	1443	-947 (-66)***
GHGI (kg CO_2_ eq. kg^-1^ grain yield)¶	0.18	0.23	0.09	0.10	0.15	0.55	0.60	0.32	0.29	0.44	-0.29 (-66)***
Barley grain yield (Mg ha^-1^)	2.81	3.52	3.45	3.22	3.25	2.75	3.35	3.45	3.65	3.30	-0.05 (-2)
Barley grain concentration (g kg^-1^)	140	151	155	142	147	145	139	148	140	143	4 (3)
Barley grain plumpness (g kg^-1^)	760	800	823	777	790	775	798	802	789	791	-1 (-1)

**Significant at *P* = 0.01

***Significant at *P* = 0.001

† Number in parenthesis denotes value in percentage.

‡ Measured at the 0- to 85-cm soil depth.

§ Global warming potential

¶ Greenhouse gas intensity

**Table 3:** Comparison of novel (no-till malt barley-pea with 134 kg N ha^-1^) and traditional (conventional till malt barley with 134 kg N ha^-1^) management practices on ecosystem services under irrigated condition in Nesson Valley, western North Dakota.

Parameter	Novel management practice					Traditional management practice					Difference†
	2008	2009	2010	2011	Mean	2008	2009	2010	2011	Mean	
Soil organic C (Mg C ha^-1^)‡	55	61	60	64	60	54	55	59	60	57	3(5)*
Soil total N (Mg N ha^-1^)‡	9.1	9.5	10.2	10.0	9.7	8.9	9.3	9.0	8.4	8.9	0.8 (9)*
Residual soil NO_3_-N (kg N ha^-1^)‡	27	34	36	39	34	30	65	87	78	65	-31 (-48)**
Total N rate (kg N ha^-1^)§	75	70	70	50	265	90	85	70	75	320	-55 (-17)
GWP (kg CO_2_ eq. ha^-1^ yr^-1^)§	745	1530	957	948	1045	1505	1950	1803	1170	1607	-562 (-35)***
GHGI (kg CO_2_ eq. kg^-1^ grain yield)¶	0.21	0.20	0.18	0.37	0.24	0.41	0.36	0.36	0.39	0.38	-0.14 (-37)***
Barley grain yield (Mg ha^-1^)	3.51	5.12	5.34	3.55	4.38	3.65	5.35	5.00	2.80	4.20	0.18 (4)
Barley grain concentration (g kg^-1^)	131	138	135	132	134	133	139	136	132	135	-1 (-1)
Barley grain plumpness (g kg^-1^)	850	875	880	843	862	842	880	828	790	835	27 (3)

*Significant at *P* = 0.05

**Significant at *P* = 0.01

***Significant at *P* = 0.001

† Number in parenthesis denotes value in percentage.

‡ Measured at the 0- to 85-cm soil depth.

§ Global warming potential

¶ Greenhouse gas intensity

In both sites, six-row malt barley and pea were planted in April every year. Conventional-tilled plots were tilled as needed and no-tilled plots were left undisturbed. Recommended rates of P (from triple superphosphate, 45% P) and K fertilizers (from muriate of potash, 60% K) were broadcast in all plots at planting and herbicides and pesticides were applied as needed to control weeds, pests, and diseases. All N fertilizer (from urea, 46% N) was broadcast at planting under non-irrigated condition in Sidney and Nesson Valley. Under irrigated condition in Nesson Valley, half of N fertilizer was broadcast at planting and other half at six weeks later. Under irrigated condition in Nesson Valley, plots were irrigated with measured amount of water according to soil water content and crop demand [30]. Malt barley and pea grain yields, protein concentration, and barley kernel plumpness were determined in August every year. Grain yields were determined from a swath of 11.0 × 1.5 m using a combine harvester after oven-drying samples at 60°C for 3 d. Samples of malt barley grain were ground to 1.0 mm and total N concentration (g N kg^-1^ plant) was determined by using the high induction furnace C and N analyzer (LECO, St. Joseph, MI). Protein concentration in malt barley grain was determined by multiplying N concentration by 6.25. Grain kernels were separated into plump, normal, and thin fractions by sieving 100 g grains in a nest of sieves containing 2.4 and 2.0 mm sieves in a strand sieve shaker (Seedburo Equipment Co., Des Plains, IL) for 3 min. Plump kernel refers to the proportion of grains retained in the 2.4 mm sieve, normal to those in the 2.0 mm sieve, and thin to those that passed through the 2.0 mm sieve. After grain harvest, crop residue was returned to the soil. For this experiment, only data from 2008 to 2011 were used.

Soil samples from 0–120 cm in Sidney and from 0–85 cm in Nesson Valley were collected after crop harvest in October from 2008 to 2011. Because of the sandy texture of the soil in the subsoil layers, we were able to sample only to a depth of 85 cm in Nesson Valley. Samples were collected from five places within the plot, composited, air-dried, and sieved to 2 mm. An additional undisturbed soil core was collected from each plot to determine bulk density, which was measured by dividing the oven-dried weight of the soil core at 105°C for 24 h by the volume of the core. The SOC and STN concentrations (g kg^-1^) in the samples were determined by using a C and N analyzer (LECO Corp., St Joseph, MI) after grinding the soil to <0.05 mm and pretreating with 6 mole L^-1^ HCl to remove inorganic C. The NO_3_-N concentration in soil samples was determined by extracting samples with 2 mole L^-1^ KCl for 1 h and analyzing the extract colorimetrically with an autoanalyzer (Lachat Instruments, Loveland, CO). The SOC, STN, and NO_3_-N contents (kg or Mg C or N ha^-1^) were determined by multiplying their concentrations by the bulk density and the thickness of the soil layer. The soil C and N sequestration rates were determined from the slope of the regression line between SOC and STN contents and year.

Static chambers were installed immediately after crop planting in 2008 to measure N_2_O and CH_4_ fluxes throughout the year until 2011 in both sites [29]. Gas samples were collected at 0, 20, and 40 min intervals in an observation date in the field and their concentrations were determined using a gas chromatograph in the laboratory. Samples were collected at 3–30 d intervals throughout the year where closer sampling (3 d) was done immediately following tillage, fertilization, planting, precipitation, irrigation, and snowmelt when gas fluxes peaked and wider sampling (7 to 30 d) in other periods when gas fluxes slowed. The fluxes of N_2_O and CH_4_ as concentrations gradient over time were calculated by using linear or nonlinear regressions and fluxes for missing dates were calculated by using linear interpolation [31, 32]. Total fluxes in a year were calculated by summing the fluxes from all observation dates. The GWP in each year was calculated by summing CO_2_ equivalents of total annual N_2_O and CH_4_ fluxes, farm operations, and N fertilization minus CO_2_ equivalent of soil C sequestration rate [6, 9, 33]. The GHGI was calculated by dividing GWP by malt barley grain yield [6].

A paired “t” test was used to compare the effect of novel vs. traditional management practice on mean SOC, STN, NO_3_-N contents, GWP, GHGI, and malt barley yield and quality in each year and across years under non-irrigated condition in Sidney and under irrigated and non-irrigated conditions in Nesson Valley [34]. Because irrigation and its interaction with management practice were significant (*P* ≤ 0.05) for all measured parameters as tested by the mixed-model of SAS [34], data were discussed separately for irrigated and non-irrigated conditions in Nesson Valley. Statistical significance was evaluated at *P* ≤ 0.05, unless otherwise stated.

## Results and Discussion

### Non-irrigated Cropping System, Eastern Montana

At the 0–120 cm depth, SOC and STN increased from 2008 to 2010 and then either remained similar or slightly decreased in 2011 with both novel and traditional management practices for non-irrigated cropping system in Sidney, eastern Montana (Table 1, S1 Table). This resulted in soil C sequestration rates of 1.8 and -3.2 Mg C ha^-1^ yr^-1^ and N sequestration rates of 0.38 and 0.28 Mg N ha^-1^ yr^-1^ at 0–120 cm for novel and traditional management practices, respectively. Mean SOC across years was 5% greater with the novel than the traditional management practice, but mean STN was not affected by management practices. Undisturbed soil, followed by continuous cropping likely increased SOC (resulting in a positive C sequestration rate) with the novel compared with the traditional management practice. In contrast, residue incorporation into the soil and aggregate disruption due to tillage, followed by increased microbial activity as a result of greater soil temperature and water content as well as absence of crops during fallow probably reduced SOC (resulting in a negative C sequestration rate) with the traditional management practice [1, 13]. Because SOC and STN are key components of soil organic matter that influences soil physical, chemical, and biological properties [1, 24, 35], increased SOC and STN with the novel management practice suggest that this practice can improve soil health and quality compared with the traditional management practice under non-irrigated or dryland cropping systems in eastern Montana.

Residual soil NO_3_-N content at 0–120 cm after crop harvest varied with years with both novel and traditional management practices (Table 1, S1 Table). This was probably due to variations in organic N mineralization from crop residue and soil as a result of differences in air temperature and precipitation as well as N removal by crops from year to year. As a result, N rate applied from N fertilizers also varied among years (Table 1). Mean residual soil NO_3_-N content across years was 21% lower with the novel than the traditional management practice. Because increased residual soil profile NO_3_-N content increases the potential for N leaching to the groundwater [3, 20], reduced NO_3_-N content suggests that the novel management practice can reduce the potential for N leaching, thereby improving water quality. Total N rate applied from 2008 to 2011, however, was 15% greater with the novel than the traditional management practice. Because applied N rate was based on soil NO_3_-N content only to a depth of 60 cm, not 120 cm, it appeared that reduced NO_3_-N content to a depth 60 cm increased N rate in the novel management practice. Mineralization of crop residue and soil organic N during tillage and fallow increases soil NO_3_-N content and reduces N fertilization rate in the traditional management practice [20, 23, 24, 26].

The GWP and GHGI also varied among years with both novel and traditional management practices (Table 1, S2 Table). Variations in N_2_O and CH_4_ fluxes due to differences in soil temperature and water content and N fertilization rates resulted in various GWP and GHGI among years, a case similar to those reported by various researchers [5, 6, 9]. Mean GWP and GHGI across years were 67 to 70% lower with the novel than the traditional management practice (Table 1). Increased C sequestration rate, followed by lower N_2_O and CH_4_ fluxes and reduced CO_2_ emissions associated with farm operations, probably decreased GWP and GHGI with the novel compared with the traditional management practice. Similar results have been reported by various researchers [6, 9, 19, 36]. This indicates that the novel management practice can reduce net GHG emissions compared with the traditional management practice in the loam soil under non-irrigated cropping systems in eastern Montana.

Malt barley grain yield, protein concentration, and grain plumpness also varied among years (Table 1, S3 Table) due to variations in air temperature and precipitation during the growing season. Mean grain yield and protein concentration across years were not significantly different between the novel and traditional management practices, and mean grain plumpness was 5% greater with the novel than the traditional management practice. The results suggest that dryland malt barley grain yield and quality can be similar or superior with the novel compared with the traditional management practice.

### Non-irrigated Cropping System, Western North Dakota

The SOC and STN at 0–85 cm increased from 2008 to 2011 for both novel and traditional management practices under non-irrigated condition in Nesson Valley, western North Dakota, except for a slight decrease in STN in 2011 for the novel management practice (Table 2, S4 Table). This resulted in C sequestration rates of 4.8 and 5.4 Mg C ha^-1^ yr^-1^ and N sequestration rates of 0.38 and 0.42 Mg N ha^-1^ yr^-1^ for novel and traditional management practices, respectively. These values were greater than those reported for SOC and STN at 0–120 cm for novel and traditional management practices under non-irrigated cropping systems in eastern Montana above. Differences in soil and climatic conditions, management practices, and depth of the soil profile may have influenced turnover rates of plant C and N to soil C and N, thereby influencing C and N sequestration rates between locations. Soil in western North Dakota was sandy loam compared to loam in eastern Montana. Western North Dakota also receives 50 mm more precipitation than eastern Montana, although air temperature is similar. Also, soils were sampled to a depth of 85 cm in western North Dakota compared to a depth of 120 cm in eastern Montana. It is likely that increased turnover rates of plant C and N into soil C and N increased C and N sequestration rates with coarse- compared with fine-textured soil. Both mean SOC and STN across years, however, were not significantly different between novel and traditional management practices.

As in eastern Montana, residual soil NO_3_-N content at 0–85 cm varied among years in the novel management practice, but increased with year in the traditional management practice (Table 2, S4 Table). Increased mineralization of crop residue and soil organic N due to tillage likely increased residual NO_3_-N from 2008 to 2011 in the traditional management practice. Mean residual soil NO_3_-N content across years was 47% lower in the novel than the traditional management practice. Similarly, total N rate applied to malt barley from 2008 to 2011 was 34% lower with the novel than the traditional management practice (Table 2). Inclusion of legumes, such as pea which fixes N from the atmosphere, in rotation with malt barley likely reduced residual soil NO_3_-N content and total N rate by supplying N from its residue in the novel management practice.

The GWP and GHGI for both management practices also varied among years (Table 2, S5 Table). The GWP and GHGI values were greater in western North Dakota than eastern Montana due to increased N_2_O and CH_4_ emissions. Both mean GWP and GHGI across years were 66% lower with the novel than the traditional management practice. Although soil C sequestration rate was slightly lower, reduced N_2_O and CH_4_ emissions, followed by decreased CO_2_ emissions associated with reduced N fertilization rate and farm operations, reduced GWP and GHGI with the novel compared with the traditional management practice.

Malt barley grain yield, protein concentration, and grain plumpness also varied among years due to variations in climatic conditions (Table 2, S6 Table). Grain yield was lower in 2008 than other years due to below-average precipitation (96 mm below the 65-yr average of 272 mm during the growing season from April to August), but rebounded in other years when growing-season precipitation was near or above the normal. Mean malt barley grain yield, protein concentration, and grain plumpness across years were not significantly different between novel and traditional management practices, suggesting that the novel management practice can produce similar levels of crop yield and quality as traditional practice, a case similar to that observed for non-irrigated cropping systems in eastern Montana.

### Irrigated Cropping System, Nesson Valley, Western North Dakota

The SOC and STN at 0–85 cm varied among years for novel and traditional management practices under irrigation condition in Nesson Valley, western North Dakota (Table 3, S4 Table). Regression analysis showed that C sequestration rates were 2.6 and 2.2 Mg C ha^-1^ and N sequestration rates 0.34 and -0.18 Mg N ha^-1^ for novel and traditional management practices, respectively. These values were lower than for the non-irrigated condition above. Leaching of water soluble C and N from the soil profile due to irrigation may have reduced SOC and STN levels, as well as C and N sequestration rates under irrigated compared with non-irrigated condition. Several researchers [37, 38, 39] also reported lower SOC under irrigated than non-irrigated cropping systems due to increased C mineralization and loss of water soluble C through leaching due to irrigation. Mean SOC and STN across years were 5 to 9% greater with the novel than the traditional management practice (Table 3). As in eastern Montana, undisturbed soil condition and reduced mineralization of crop residue and soil organic matter may have increased SOC and STN in the novel compared with the traditional management practice.

Residual soil NO_3_-N content at 0–85 cm also varied among years (Table 3, S5 Table). These values were greater than those observed for the non-irrigated condition above, probably the results of increased crop residue and soil organic N mineralization as well as greater N rate applied to irrigated barley. Crops can utilize only 50 to 70% of applied N, and increased N rate can increase residual soil profile NO_3_-N content [40, 41]. Mean residual NO_3_-N content was 31% lower with the novel than the traditional management practice, a value similar to that obtained for non-irrigated condition. Because of variations in residual soil NO_3_-N content, N rate applied to malt barley also varied among years (Table 3). Total N rate from 2008 to 2011 was 17% lower in the novel than the traditional management practice, which is half of that obtained in the non-irrigated condition. Although N supplied from pea residue reduced N rate in the novel compared with the traditional management practice under both irrigated and non-irrigated conditions, lower N rate under the irrigated condition was due to greater residual soil NO_3_-N content.

As with other sites, GWP and GHGI varied among years in both management practices (Table 3) due to variations in N_2_O and CH_4_ emissions and N fertilization rates. These values were greater than those observed under the non-irrigated condition (Table 2), probably due to enhanced microbial activity as a result of increased soil water availability from irrigation and N substrate availability from N fertilization. Mean GWP and GHGI across years were 35 to 37% lower with the novel than the traditional management practice. Increased C sequestration rate, followed by lower N_2_O and CH_4_ emissions and reduced CO_2_ emissions associated with N fertilization and farm operations reduced GWP and GHGI with the novel compared with the traditional management practice.

Malt barley grain yield, protein concentration, and grain plumpness also varied among years due to differences in growing season climatic conditions (Table 3, S6 Table). Grain yield and grain plumpness were higher with the irrigated than the non-irrigated condition, but protein concentration was similar (Tables 2 and 3). Increased soil water availability due to irrigation probably increased grain yield and grain plumpness under the irrigated condition. As with the non-irrigated condition, mean grain yield, protein concentration, and grain plumpness across years were not significantly different between novel and traditional management practices (Table 3). Mean malt barley protein concentration and grain plumpness across years obtained in this study were within the acceptable levels of <135 g kg^-1^ protein concentration and >800 g kg^-1^ grain plumpness for six-row malt barley for malting purpose [42].

## Conclusions

The SOC and STN at 0–120 cm increased, but residual soil NO_3_-N content, N rate, GWP, GHGI, malt barley grain yield, protein concentration, and grain plumpness varied among years from 2008 to 2011 in both novel and traditional management practices under irrigated and non-irrigated conditions in eastern Montana and western North Dakota. Mean SOC, STN, malt barley grain yield, protein concentration, and grain plumpness across years were greater or similar, but residual soil NO_3_-N content, N rate, GWP, and GHGI were lower with the novel than the traditional management practice. These results suggest that the novel management practice, such as no-till malt barley-pea with reduced N rate, can improve soil and environmental quality and sustain malt barley yield simultaneously by increasing soil C and N storage, reducing the potential for N leaching and net GHG emissions, and maintaining malt barley yield and quality compared with the traditional practices, such as conventional till continuous malt barley or malt barley-fallow with recommended N rates under irrigated and non-irrigated cropping systems in the northern Great Plains. The practice may also reduce agricultural inputs, such as the energy requirement for tillage and N fertilizer. Another benefit would be reduced incidences with weeds, diseases, and pests due to crop rotation in the novel management practice compared with monocropping or crop-fallow in the traditional management practice. The results can be applied to other regions with similar soil and climatic conditions where small grains are grown.

## Supporting Information

S1 Table Soil C and N data for non-irrigated cropping system in eastern Montana. (XLSX)

S2 Table Data for global warming potential and greenhouse intensity for non-irrigated cropping system in eastern Montana. (XLSX)

S3 Table Data for malt barley yield, grain protein concentration, and grain plumpness for non-irrigated cropping system in eastern Montana. (XLSX)

S4 Table Soil C and N data for non-irrigated and irrigated cropping systems in western North Dakota. (XLSX)

S5 Table Data for global warming potential and greenhouse intensity for non-irrigated and irrigated cropping systems in western North Dakota. (XLSX)

S6 Table Data for malt barley yield, grain protein concentration, and grain plumpness for non-irrigated and irrigated cropping systems in western North Dakota. (XLSX)

## Abbreviations

GHG: greenhouse gas
GHGI: greenhouse gas intensity
GWP: global warming potential
SOC: soil organic C
STN: soil total N
